# Coenzyme Q10 for Acute Stanford Type A Aortic Dissection: Protocol for a Prospective, Randomized, Double-Blind, Placebo-Controlled, Single-Center Feasibility Trial

**DOI:** 10.3390/jcm15208103

**Published:** 2026-10-19

**Authors:** Lu Dai, Shi-Pan Wang, Yin-Fan Zhu, Hao-Tian Wu, Bao-Fu Zheng, Hai-Yang Li, Wen-Jian Jiang, Hong-Jia Zhang

**Affiliations:** 1 Beijing Anzhen Hospital, Capital Medical University, Beijing 100029, China; 2 Department of Epidemiology, Beijing Institute of Heart, Lung and Blood Vessel Diseases, Beijing 100029, China

**Keywords:** coenzyme Q10, acute type A aortic dissection, Stanford type A aortic dissection, feasibility trial, randomized controlled trial, placebo, SPIRIT 2025

## Abstract

**Background/Objectives:** Acute Stanford type A aortic dissection (TAAD) is a catastrophic cardiovascular emergency. Emergency surgery is the standard life-saving treatment, but some patients decline operative repair, and no established pharmacological therapy has been shown to stabilize acute TAAD. Preclinical work by our team implicates mitochondrial dysfunction and smooth muscle cell phenotypic switching in aortic dissection and suggests that coenzyme Q10 may reduce dissection-related injury. This study aims to determine the feasibility of a future definitive randomized trial and to obtain preliminary safety and exploratory efficacy information. **Methods:** This prospective, randomized, double-blind, placebo-controlled, single-center feasibility trial will enroll 60 adults with CTA-confirmed Stanford type A aortic dissection within 24 h of symptom onset who decline emergency surgical repair after standard surgical counseling. Participants will be randomized 1:1 to oral coenzyme Q10 100 mg three times daily or matched placebo for 14 days, in addition to the same guideline-directed supportive medical care. The primary feasibility outcome is recruitment feasibility, defined as randomization of 60 participants within 12 months after recruitment initiation. Secondary feasibility outcomes include treatment adherence, follow-up completion, dropout, missingness, outcome-assessment feasibility, and candidate endpoint event rates. The primary exploratory clinical outcome is 14-day all-cause mortality; additional exploratory outcomes include aortic rupture death, false-lumen thrombosis, cardiac tamponade, worsening pericardial effusion, and organ malperfusion syndromes. Safety outcomes include adverse events, serious adverse events, adverse drug reactions, suspected unexpected serious adverse reactions, and prespecified laboratory indices. **Results:** The trial is designed to establish whether recruitment, blinded treatment delivery, follow-up, outcome collection, and safety monitoring are operationally feasible and to provide estimates for planning a larger confirmatory trial. **Conclusions:** This feasibility trial will inform the design and conduct of future definitive evaluation of coenzyme Q10 in acute TAAD. Trial registration: Chinese Clinical Trial Registry, ChiCTR2600123230.

## 1. Introduction

Aortic dissection (AD) is a life-threatening cardiovascular disorder characterized by a tear within the aortic wall and the formation of a false lumen, with the potential for rupture and death; malperfusion of branch vessels may also lead to ischemic organ injury and poor outcomes [1,2]. The Stanford classification divides AD into type A and type B according to whether the ascending aorta is involved [3]. Acute Stanford type A aortic dissection (TAAD) carries particularly high early mortality, with historical data indicating mortality of approximately 50% within 48 h, 70% within 2 weeks, and up to 90% within 1 month if untreated [4,5]. Although AD is often considered in the differential diagnosis of acute chest pain together with acute coronary syndrome and pulmonary embolism, its early risk of death is exceptionally high [6,7].

Emergency surgical repair is the current standard treatment for acute TAAD [1]. However, operative mortality remains substantial; data from the International Registry of Acute Aortic Dissection suggest a short-term surgical mortality approaching 20% in contemporary practice [8]. In addition, because TAAD surgery is complex and concentrated in specialized centers, many patients require urgent transfer, and delayed access to definitive care remains an important clinical challenge [9]. For patients who refuse surgery or cannot undergo immediate operative repair, there is an unmet need for pharmacological strategies that may stabilize the dissected aorta and improve early survival.

The natural history of AD is time-dependent. Current aortic guidelines classify AD according to the interval from symptom onset as hyperacute (<24 h), acute (1–14 days), subacute (15–90 days), and chronic (>90 days) [1]. Clinical observations suggest that mortality is highest in the hyperacute and acute phases and decreases as the disease evolves into the subacute and chronic phases [10]. Similar time-dependent differences have been reported among surgically treated TAAD patients: patients undergoing surgery in the acute phase have higher early and mid-term mortality than those treated in the subacute or chronic phase, while outcomes for subacute/chronic TAAD may approximate those for elective thoracic aortic aneurysm surgery [10,11,12,13]. These findings imply that the aortic wall may undergo biological changes over time that reduce rupture risk and improve operative safety.

Coenzyme Q10 is a lipid-soluble quinone involved in the mitochondrial electron transport chain and oxidative phosphorylation. It has a long history of human use, is marketed as a nutritional supplement in many countries, and is available as a pharmaceutical product in China. Previous studies and reviews have evaluated coenzyme Q10 in conditions related to mitochondrial dysfunction and cardiovascular disease, and the available evidence suggests an overall favorable safety and tolerability profile, including experience with doses higher than those used in routine approved indications [14,15].

The study team previously investigated ascending aortic tissues from control donors and patients with acute, subacute, and chronic TAAD using single-cell transcriptomic and experimental approaches [16]. This work showed that synthetic smooth muscle cells increase in TAAD and that mitochondrial oxidative phosphorylation in smooth muscle cells is impaired. In an experimental aortic dissection model, coenzyme Q10 reduced TAAD incidence, rupture-related mortality, and aortic diameter, and it helped maintain the contractile smooth muscle cell phenotype [16]. These findings provide the biological rationale for testing whether coenzyme Q10 can help patients with acute TAAD safely transition through the highest-risk early phase, particularly among patients who decline surgical repair.

### 1.1. Rationale for the Comparator

A matched placebo is used because no established active pharmacological comparator exists for stabilizing acute TAAD in patients who decline surgical repair. Placebo control, in addition to the same guideline-directed supportive medical care in both groups, allows for preliminary estimation of whether coenzyme Q10 produces clinically meaningful differences in candidate efficacy and safety outcomes while maintaining blinding.

### 1.2. Objectives

The primary objective is to determine the recruitment feasibility of a future confirmatory randomized controlled trial of coenzyme Q10 for acute TAAD, using achievement of the planned recruitment target as the primary feasibility criterion. Additional feasibility objectives include assessment of randomization, blinded treatment delivery, follow-up, treatment adherence, outcome-assessment time points, missing data, and endpoint selection. The primary exploratory efficacy outcome is 14-day all-cause mortality; other exploratory outcomes include aortic rupture death, false-lumen thrombosis, cardiac tamponade, worsening pericardial effusion, organ malperfusion syndromes, and safety outcomes.

## 2. Materials and Methods

### 2.1. Study Design, Registration, and Trial Administration

This is a prospective, randomized, double-blind, placebo-controlled, single-center, small-sample feasibility trial with two parallel groups. Eligible participants will be randomized 1:1 to coenzyme Q10 or matching placebo. The trial is exploratory/feasibility in its framework and is not powered to test a definitive superiority hypothesis. The protocol has been prepared in accordance with the SPIRIT 2025 statement [17] and the completed SPIRIT 2025 checklist is provided in Supplementary Table S1.

The protocol identifier is AZYYIIT-ZHJ-001. The trial has been prospectively registered in the Chinese Clinical Trial Registry (ChiCTR2600123230; registration date: 23 April 2026). Research ethics approval has been obtained from the Ethics Committee of Beijing Anzhen Hospital, Capital Medical University (approval No. 2026-07).

This is an investigator-initiated clinical study initiated and conducted by Beijing Anzhen Hospital, Capital Medical University. Prof. Hongjia Zhang is the principal investigator. No commercial entity acts as the sponsor/initiator. Contracted commercial organizations may provide the operational services described below, but they do not act as the study sponsor/initiator or have authority over the scientific design, statistical interpretation, or publication of the trial.

The study is investigator-initiated. The principal investigator and study team are responsible for trial design, clinical conduct, safety reporting, interpretation, and manuscript preparation. Certain operational functions are delegated to contracted service providers, including EDC system provision and maintenance, data management, monitoring, pharmacovigilance support, project management, and SMO services. The statistical analysis will be performed by the Department of Epidemiology, Beijing Institute of Heart, Lung and Blood Vessel Diseases. Any funding source, study-drug support, and operational role will be fully disclosed before submission.

The trial team includes the principal investigator, key sub-investigator(s), sub-investigators, investigators, research nurses, drug administrator(s), EDC administrator(s), clinical research coordinator(s) (CRCs), clinical research associate(s) (CRAs), CRO staff, SMO staff, and a statistical team. Yidixi Pharmaceutical Technology (Jiaxing) Co., Ltd. Jiaxing, China will provide CRO services, including Clinflash EDC system deployment, validation and maintenance, data management, monitoring by CRAs, pharmacovigilance support, and project management. Shanghai Zhaohui Medical Technology Co., Ltd. Shanghai, China will provide SMO services. The hospital clinical research management committee may conduct additional oversight.

The full protocol and statistical analysis plan will be available from the corresponding author upon reasonable request and may be uploaded to the trial registry, subject to institutional and ethics requirements.

### 2.2. Study Setting and Participants

The trial will be conducted at Beijing Anzhen Hospital, Capital Medical University, Beijing, China, a tertiary hospital with a complete clinical research management system and expertise in the diagnosis and management of acute aortic disease. Potentially eligible patients will be identified in the emergency center after project approval, ethics approval, registration, and completion of the study initiation meeting.

#### 2.2.1. Eligibility Criteria

The inclusion and exclusion criteria for trial participants are summarized in Table 1.

#### 2.2.2. Eligibility of Sites and Intervention Deliverers

Because this is a single-center trial, there are no site-level eligibility criteria beyond conduct at Beijing Anzhen Hospital. Study physicians, research nurses, drug administrators, EDC administrators, CRCs, CRAs, CRO staff, SMO staff, and other personnel involved in trial procedures must complete Good Clinical Practice training and study-specific protocol training before participating in the trial.

### 2.3. Ethics and Informed Consent

The trial protocol and informed-consent materials have received research ethics approval from the Ethics Committee of Beijing Anzhen Hospital, Capital Medical University (approval No. 2026-07). Before any study-specific procedures, a trained investigator will provide the patient or legal representative with the ethics-approved written participant information, explain the study orally, and answer any questions. The consent discussion will include the study purpose, intervention, randomization, blinding, potential benefits and risks, alternative treatments including surgery and standard medical care, privacy protections, voluntary participation, and the right to withdraw. Written informed consent will be obtained from the patient when the patient has full civil capacity and decision-making ability. When the patient lacks capacity, written consent will be obtained from the legally authorized representative in accordance with applicable regulations. If the patient or legal representative has impaired reading ability, an impartial witness will be present throughout the consent process and will sign and date the consent form. After written informed consent is completed, the CRC will register participant information in the EDC system and assign a screening number. Consent will also cover the review of medical records and collection of study data; any additional use of residual samples or data for future research will require appropriate consent and ethics approval.

Before research consent is discussed, each potentially eligible patient will first be evaluated by the attending aortic surgical team, and emergency surgical repair will be clearly explained as the standard-of-care treatment. The decision to decline surgery will be made independently of trial participation and documented in the source medical record together with the stated reason(s) for refusal and the patient’s decision-making capacity; use of a legally authorized representative will also be recorded when applicable. If a participant or representative subsequently reconsiders surgery, trial participation will not delay or restrict definitive treatment: the patient will undergo prompt surgical reassessment, study medication will be discontinued when clinically appropriate, and the timing and reason for crossover to surgery will be documented.

The trial targets patients with acute TAAD who decline surgical treatment, a group with extremely high risk and limited evidence-based medical options. Coenzyme Q10 has extensive prior human exposure and a favorable general safety profile, but the proposed dose exceeds the domestic label dose, and the indication is off-label. The trial therefore includes strict eligibility criteria excluding patients in irreversible organ failure or extreme instability, detailed safety assessments, active monitoring for AE/SAE/ADR/SUSAR, predefined discontinuation criteria, and prompt treatment of complications. All participants will receive guideline-directed supportive care regardless of treatment assignment.

### 2.4. Randomization, Allocation Concealment, and Blinding

#### 2.4.1. Sequence Generation

The randomization workflow comprises two distinct steps. First, an independent statistician who is not involved in trial implementation will generate blinded drug numbers (D-0XX) for coenzyme Q10 and the placebo in a 1:1 ratio using R software (version 4.3), with dynamic permuted blocks of 4 or 6 for drug preparation and inventory. Second, after an eligible participant is enrolled, participant-level treatment allocation will be performed centrally through the interactive response technology (IRT) system using minimization with sex (male/female) as the balancing factor and an 80% biased allocation probability. The IRT system will then return an available drug number corresponding to the assigned treatment. Thus, block randomization is used for the generation of the blinded drug-number pool, whereas participant treatment allocation is governed by the minimization algorithm. Allocation information will remain concealed from participants, investigators, treating clinicians, outcome assessors, and blinded statisticians until the prespecified unblinding procedures are completed.

#### 2.4.2. Allocation Concealment and Implementation

Allocation will be implemented through the IRT system integrated with the EDC workflow. After informed consent and eligibility confirmation during screening, the CRC will register participant information in the EDC system and assign a screening number. When enrolment is completed, the PI or key sub-investigator will use the IRT system to assign the registration/randomization number and corresponding drug number. Study medication packages corresponding to the assigned drug number will be dispensed without revealing the group assignment. Active and placebo tablets will be identical in packaging, appearance, smell, and administration schedule.

Investigators and research nurses will identify and screen potential participants. The CRC will enter screening information in the EDC system. After eligibility is confirmed and enrolment is completed, the PI or key sub-investigator will obtain the registration/randomization number and corresponding drug number through the IRT system. The drug administrator will transfer the assigned medication to the research nurse, and the investigator will issue the research medication order. The research nurse will check the order and administer the study medication. Personnel involved in enrolment, treatment, clinical care, outcome assessment, and data analysis will not have access to the allocation sequence during the blinded phase, except under emergency unblinding procedures.

#### 2.4.3. Blinding and Unblinding

Participants, treating clinicians, investigators, research nurses, outcome assessors, data managers, CRCs, CRAs, and statisticians involved in blinded analysis will remain blinded to treatment assignment until the prespecified unblinding steps are completed. Blinding will be achieved by the use of matched active and placebo tablets with identical size, shape, color, appearance, odor, packaging, dose, frequency, and route of administration. Drug-number coding and the code-to-treatment correspondence will be prepared by the independent statistician and provided to the drug packaging company; the corresponding drug transfer information will be entered into the IRT system. Personnel involved in trial conduct will not have access to the blind list during the blinded phase.

After all CRFs have been entered into the EDC and checked, a blinded data review will be conducted to evaluate study completion, dropout, eligibility, completeness, logic consistency, outliers, visit windows, concomitant medications, and AEs. When all data are collected, queries resolved, and analysis populations defined, the database will be locked. The first unblinding will reveal coded treatment groups (e.g., group A and group B) to the statistician for analysis without identifying active or placebo treatment. The second unblinding will occur at the summary meeting after the statistical analysis report is completed. Emergency unblinding is permissible only when knowledge of the assigned intervention is essential for urgent clinical management, such as an SAE, severe interference, or clinical worsening requiring emergency measures. Only the principal investigator may authorize emergency unblinding, which will be performed online and documented with the date, reason, and supporting records.

### 2.5. Intervention and Comparator

An independent statistician not involved in trial implementation will assign drug numbers and provide the drug-number-to-treatment-code correspondence to Beijing Fuyuan Pharmaceutical Co., Ltd Beijing, China. Active coenzyme Q10 and matched placebo will be packaged and labeled according to these drug numbers so that treatment assignments remain indistinguishable. Information on drug-number coding and drug transfer orders will be entered into the IRT system for subsequent participant allocation. Study medication will be stored under light-protected, sealed, and dry conditions according to product requirements. Empty packages will be counted and documented by the drug administrator and disposed of according to hospital procedures. The interventions, administration schedules, and concomitant care for both treatment groups are summarized in Table 2.

#### 2.5.1. Discontinuation or Modification of Allocated Intervention

Participants may withdraw from the study at any time. Study medication will be discontinued when continued administration is not clinically appropriate, including a confirmed allergy to coenzyme Q10, a serious adverse event or suspected unexpected serious adverse reaction judged to require discontinuation, clinically important deterioration for which the investigator believes trial treatment should stop, participant or legal representative request, or other serious protocol-defined safety concerns. Dose reduction is not planned. Participants who discontinue study medication will receive appropriate medical care and, whenever possible, continue outcome and safety follow-up according to the protocol.

#### 2.5.2. Treatment Adherence

Because participants will be hospitalized during the acute treatment period whenever clinically indicated, medication will be administered under medical or nursing supervision according to research orders. Administration will be documented in study records and the EDC. Empty packages will be counted and recorded. Missed doses, incorrect doses, or administration outside the planned schedule will be documented as protocol deviations when applicable.

#### 2.5.3. Concomitant Care

All participants will receive the same guideline-directed supportive and symptomatic treatment for acute aortic dissection, including heart-rate control, blood-pressure control, analgesia, sedation when clinically indicated, and treatment of comorbidities and complications. Consistent with contemporary aortic-disease guidance, clinicians will generally aim for a systolic blood pressure below 120 mmHg or the lowest pressure that maintains adequate end-organ perfusion, as well as a heart rate of approximately 60–80 beats/min when tolerated; these are clinical treatment goals rather than rigid trial-mandated thresholds and may be individualized according to hemodynamic status and malperfusion. Intravenous beta-blockade is generally preferred for initial anti-impulse therapy when not contraindicated, with alternative rate-control agents and vasodilators used as clinically appropriate. Opioid analgesia and sedation may be used when necessary to control pain or sympathetic activation. Anticoagulants and antiplatelet agents are not prohibited by the trial and may be used when clinically justified for another indication. All concomitant medications will be recorded with the drug name, indication, dose, frequency or infusion rate, route, start date, current treatment status, and stop date. No additional trial-specific imaging triggers are imposed; unscheduled CTA or echocardiography will be obtained when clinically indicated. Protocol deviations will be documented and managed as described in Section 2.12.2.

### 2.6. Outcomes and Harms

The study outcomes are categorized into primary and secondary feasibility outcomes, exploratory efficacy outcomes, and safety outcomes. Their definitions and assessment time points are summarized in Table 3.

#### 2.6.1. Outcome Assessment and Imaging Definitions

Scheduled CTA and echocardiography will be performed according to the participant timeline using the hospital’s routine acute-aortic-syndrome imaging protocols; no additional research-specific acquisition parameters are imposed. In accordance with the protocol and the version 2.0 CRF, false-lumen status will be recorded by aortic segment as false-lumen disappearance, complete thrombosis, partial thrombosis, no thrombosis, or not involved at baseline. For the prespecified exploratory endpoint, false-lumen thrombosis is defined as partial or complete thrombosis in any aortic segment involved by dissection. For reporting purposes, partial thrombosis indicates coexistence of contrast-opacified patent lumen and thrombus within the false lumen of the same segment, whereas complete thrombosis indicates absence of contrast opacification with thrombus occupying the false lumen of that segment.

Pericardial effusion will be assessed by echocardiography and categorized by the maximal end-diastolic echo-free space as mild (<10 mm), moderate (10–20 mm), or large (>20 mm). Worsening pericardial effusion is defined as a new effusion or an increase compared with baseline; no additional absolute-change threshold is introduced. Cardiac tamponade requires compatible clinical manifestations together with at least one prespecified echocardiographic feature, including right-sided chamber collapse, abnormal inferior vena cava findings, swinging heart, pericardial effusion, marked respiratory variation in transvalvular flow, abnormal hepatic venous flow, or septal shift. Organ malperfusion requires branch-vessel involvement on CTA together with the organ-specific clinical, laboratory, or imaging findings prespecified in the protocol.

Imaging outcome assessors will remain blinded to treatment allocation. CTA and echocardiography are scheduled at screening and on Study Days 7 and 14; additional imaging may be obtained whenever clinically indicated. If death, crossover to surgery, or another major clinical event precludes a scheduled imaging assessment, the corresponding imaging endpoint will be recorded as unavailable rather than imputed as absent.

#### 2.6.2. Harms

An AE is any unfavorable medical event occurring from the time of informed-consent signing, whether or not causally related to study medication. Abnormal laboratory values will be reported as AEs if they are accompanied by symptoms, require additional diagnostic testing or medical/surgical intervention, lead to study-drug modification outside protocol expectations, or are judged by the investigator to constitute an AE. Baseline medical conditions present at consent will not be recorded as AEs unless they worsen, develop complications, or increase in frequency during the study.

AE severity will be graded from 1 to 5: grade 1, mild; grade 2, moderate; grade 3, severe or medically significant but not immediately life threatening; grade 4, life threatening requiring urgent intervention; and grade 5, AE-related death. Causality will be assessed as definitely related, probably related, possibly related, possibly unrelated, pending evaluation, or not assessable. Events assessed as definitely, probably, possibly related, or not assessable will be recorded as ADRs, using a conservative principle when information is incomplete. SAEs and SUSARs will be reported according to applicable Chinese GCP and safety-reporting requirements. SAEs will be reported to the principal investigator within 24 h of investigator awareness; SUSARs will be submitted to the ethics committee within regulatory timelines.

### 2.7. Participant Timeline

X indicates that the procedure is scheduled; (X) indicates as needed or if clinically available. Study Day 0 is the registration/randomization day and the first day of study medication. To harmonize terminology throughout the manuscript, treatment Day 2 corresponds to Study Day 1, treatment Day 3 to Study Day 2, treatment Day 8 to Study Day 7, and treatment Day 15 to Study Day 14. The final Study Day 14 visit has an allowable window of +2 days. The schedule of enrolment, study intervention, clinical assessments, and follow-up is presented in Table 4.

### 2.8. Sample Size and Recruitment

#### 2.8.1. Sample Size

This feasibility trial is not powered to test a confirmatory efficacy hypothesis. For planning purposes, 14-day all-cause mortality is the primary clinical endpoint for a future definitive trial and uses a hypothetical mortality rate of 50.0% in the placebo group and 30.0% in the coenzyme Q10 group, with a two-sided alpha of 0.05 and 80% power. Under these assumptions, PASS 15.0 estimated that 93 participants per group (186 total) would be required; allowing 10% dropout would require 104 participants per group (208 total). The assumed 30.0% mortality rate in the coenzyme Q10 group is a planning assumption rather than an evidence-based expected treatment effect because no human efficacy data for coenzyme Q10 in acute TAAD are currently available. The present study is feasibility-oriented and is not intended to establish statistically significant between-group efficacy differences; any exploratory comparison will be interpreted as hypothesis-generating. The final feasibility sample size is 60 participants, randomized 1:1 with 30 participants per group, approximately 20–30% of the projected definitive-trial size. The definitive-trial sample size will be recalculated using event rates, treatment-effect estimates, and confidence intervals observed in the feasibility study together with a clinically meaningful target effect.

#### 2.8.2. Recruitment

After institutional and ethics approvals are obtained and the study initiation meeting has been completed, a research nurse will be stationed at the triage desk of the emergency center to identify patients with suspected or confirmed TAAD. Investigators will reassess potentially eligible patients, confirm diagnosis and timing, evaluate willingness to participate after surgical treatment has been declined, and screen eligibility using objective inclusion and exclusion criteria. After informed consent, the CRC will register the patient in the EDC system and assign a screening number. If the patient remains eligible and continues to agree to trial participation, enrolment will be completed in the EDC system, and the PI or key sub-investigator will use the IRT system to assign the registration/randomization number and corresponding drug number. Recruitment is planned to continue for up to 1 year or until 60 participants have been randomized, whichever occurs first.

The current protocol is version V2.0, dated 9 June 2026. Version V2.0 was developed from the initial V1.0 protocol to incorporate operational refinements, including the IRT-supported randomization workflow, CRO/SMO participation, and expanded EDC/data-management procedures, while preserving the core population, intervention, comparator, sample size, and follow-up framework. The present manuscript reflects version V2.0. Any further substantive amendment affecting trial conduct will undergo the required ethics review and trial registry updating before implementation.

### 2.9. Data Collection, Retention, and Management

#### 2.9.1. Data Collection

Trial data will be collected from source medical records, protocol-specific assessments, imaging reports, laboratory reports, medication records, AE/SAE reports, and follow-up records. Prespecified enrolment data will also include the stated reason(s) for declining surgery, decision-making capacity at consent, use of a legally authorized representative where applicable, and any subsequent reconsideration of surgery or crossover to operative treatment, including timing and reason. Screening echocardiography and laboratory data must be in-hospital results obtained after first symptom onset and before the start of study treatment; if multiple eligible results exist, the last pre-treatment result will be used. Follow-up CTA, echocardiography, and laboratory data may be based on in-hospital results after the treatment period; if multiple eligible results exist, the last pre-discharge or protocol-relevant result will be used.

Prespecified laboratory measures include hematology (white blood cell count, neutrophil percentage, neutrophil count, red blood cell count, hemoglobin, platelet count, and lymphocyte count), biochemistry (alanine aminotransferase, aspartate aminotransferase, total bilirubin, direct bilirubin, blood urea nitrogen, creatinine, and C-reactive protein), coagulation (activated partial thromboplastin time, prothrombin time, fibrinogen degradation products, and D-dimer), thrombosis markers (thrombin–antithrombin complex, plasmin–antiplasmin complex, tissue plasminogen activator–plasminogen activator inhibitor complex, and thrombomodulin antigen), myocardial injury markers (cardiac troponin, myoglobin, and CK-MB), blood gas analysis (PaO_2_, PaCO_2_, and lactate), and beta-human chorionic gonadotropin for women of childbearing potential.

#### 2.9.2. Retention and Follow-Up

The acute nature of TAAD, high early event risk, and short intervention window support intensive inpatient follow-up whenever feasible. The protocol-defined research follow-up ends at Study Day 14 (+2 days) because the purpose of this feasibility study is to evaluate recruitment, delivery of a 14-day blinded intervention, acute safety, and feasibility of candidate endpoints during the hyperacute/acute phase rather than sustained survival or late aortic remodeling. Longer-term survival, aortic remodeling, late safety, and recovery outcomes will be considered in the subsequent definitive trial, while routine clinical follow-up will continue according to standard care. Research staff will maintain contact with participants and/or legal representatives, document contact information, and collect survival, outcome, safety, and concomitant medication information until the end of the protocol-defined follow-up. For participants who withdraw early, efficacy outcomes will be collected until the day of withdrawal. AE/SAE and concomitant medication data will be collected until Day 14 if withdrawal occurs more than 7 days before the planned end of treatment; if withdrawal occurs within 7 days before the planned end of treatment, these data will be collected until 7 days after withdrawal, where feasible.

#### 2.9.3. Data Management

The Clinflash EDC system provided by Yidixi Pharmaceutical Technology (Jiaxing) Co., Ltd. (Jiaxing, China) will be used for trial data collection and management. The system has undergone computerized-system validation and is intended to comply with the 2020 Chinese Good Clinical Practice requirements and technical guidance for clinical-trial data management. The EDC system will support screening numbers, randomization numbers, role-based access, audit trails, data validation, and data backup. Data may be entered directly into the EDC as source data or transcribed from paper CRFs into the EDC. Investigators are responsible for the authenticity, completeness, and quality of entered data. Printed CRFs, where generated, will be signed by the investigator and archived as source documents. The CRO/EDC service provider will design and configure the database, develop edit checks and logic-checking rules, update the EDC configuration after protocol amendments, provide system maintenance and 24 h technical support, train users before system launch, perform system and manual data checks, issue queries, and provide regular data-management reports to the PI.

Data validation will include real-time system warnings for abnormal entries and manual logic checks by the EDC administrator. Queries will be issued through the EDC, and investigators must respond to each query by correcting erroneous data or explaining why the entry is accurate. CRCs will remind and assist investigators in timely query resolution. The EDC administrator will ensure that query records and responses are traceable and tamper-resistant and will provide a complete query-management report after database lock. After all data are entered and queries resolved, the database will be soft-locked. A third-party statistician will generate a data-review report for final team review. The database will be formally locked after signatures by the principal investigator, EDC administrator, and third-party statistical lead. After database lock, the EDC administrator will provide the PI with a complete backup of the locked database, including data files, audit trails, query-management records, and relevant export files. Locked data will not be modified unless a critical error affecting efficacy or safety outcomes is confirmed and formally approved by the same responsible personnel. The EDC administrator will archive EDC system data and related documents, including database design documents, validation documents, user training records, data-entry records, audit trails, query-management records, lock/unlock records, and data-export records.

### 2.10. Statistical Analysis

The statistical analysis will be primarily descriptive and will focus on feasibility, safety, and exploratory efficacy. Analyses will be conducted after data cleaning and database lock. Categorical variables will be summarized as counts and percentages. Continuous variables will be assessed for distributional characteristics and summarized as the mean and standard deviation or median and interquartile range, as appropriate. Exploratory between-group comparisons may be performed using chi-square or Fisher’s exact tests for categorical variables and appropriate parametric or non-parametric tests for continuous variables, using two-sided tests with alpha = 0.05. Because this is a feasibility study, these *p* values will be considered hypothesis-generating only and will not be used to establish treatment efficacy. Effect estimates, including absolute risk differences and risk ratios with 95% confidence intervals where estimable, will be emphasized to inform the design of a future definitive trial.

The primary feasibility outcome will be reported as whether the target of 60 randomized participants within 12 months was achieved, together with the numbers screened, eligible, consented, and randomized and the observed recruitment rate. Secondary feasibility metrics, including treatment adherence, protocol completion, follow-up completion, dropout, missingness, blinded treatment delivery, and endpoint-assessment feasibility, will be summarized quantitatively. These feasibility measures will instead be interpreted collectively to identify modifications required before a definitive trial. Missing data will not be imputed in the primary feasibility analyses; the amount and pattern of missingness will be summarized. Analysis populations, including randomized, treated, and per-protocol/feasibility populations, will be finalized during blinded data review before unblinding.

### 2.11. Monitoring, Safety Oversight, and Stopping Rules

#### 2.11.1. Trial Monitoring

Yidixi Pharmaceutical Technology (Jiaxing) Co., Ltd., as the contracted CRO, will appoint CRAs to provide clinical monitoring services. Monitoring will include, but will not be limited to, protocol-compliance review, source-data verification, consistency checks between EDC data and source documents, and review of SAE reporting timeliness. All personnel involved in the trial must complete GCP and protocol-specific training. The hospital clinical research management committee may appoint monitors to conduct unscheduled on-site or telephone monitoring. Structured templates will be used for source documentation to reduce incomplete or heterogeneous recording. Investigators and CRCs will complete CRFs truthfully, completely, and accurately according to CRF completion requirements. Data-management procedures will be applied throughout the clinical and data-processing phases.

#### 2.11.2. Interim Analyses and Stopping Rules

No formal interim efficacy analysis is planned because this is a small feasibility trial. The trial may be suspended or terminated if requested by the principal investigator, national or municipal health authorities, the hospital clinical research management committee, or the ethics committee; if legal, ethical, or research-integrity violations occur; if a serious study-drug quality defect is identified; if AEs/SAEs create major safety concerns; if improper financial or other conflicts emerge; if existing data demonstrate a marked treatment difference before target recruitment; if new higher-level evidence definitively answers the study question; if recruitment is too slow to complete; or if funding is insufficient to continue. If the study is suspended or terminated, investigators will summarize enrolment, dropout, completion, AE/SAE counts, reasons for termination, notification plans, ethics reporting, and clinical follow-up for active participants.

#### 2.11.3. Harms Reporting and Safety Oversight

All AEs occurring from the time of informed-consent signing will be treated promptly after the investigator becomes aware of them. Investigators will collect original medical records and the test results needed for AE documentation. AE reports will include the event name, brief description, start and end dates, severity grade, causality assessment, action taken with study drug, treatment for the AE, outcome, and whether the event is serious. Medical diagnoses will be recorded preferentially rather than isolated symptoms or signs when a diagnosis is available. AEs will be followed until resolution, stabilization, return to baseline, attribution to non-study causes, loss of ability to obtain further information despite reasonable efforts, or the protocol-defined early-withdrawal follow-up cutoff.

SAEs occurring from the time of informed-consent signing will be handled immediately and reported to the principal investigator within 24 h of investigator awareness. The study team, with pharmacovigilance support from the CRO where applicable, will assess whether an SAE is an ADR and whether it meets SUSAR criteria. Fatal or life-threatening SUSARs will be reported to the ethics committee within 7 days of first awareness with follow-up within the subsequent 8 days. Non-fatal and non-life-threatening SUSARs will be reported within 15 days. Additional follow-up reports will be submitted within 15 days after new information becomes available.

### 2.12. Confidentiality, Protocol Amendments, and Dissemination

#### 2.12.1. Confidentiality

Participant privacy will be protected throughout the trial. External transfers or submissions of study materials will identify participants only by registration number and initials rather than full name or personal identification number. Paper records will be stored in locked cabinets, and electronic data will be accessible only to authorized users through password-protected accounts with role-based permissions. EDC data will be backed up to prevent loss. All personnel with access to study data must sign confidentiality agreements; confidentiality obligations will continue after study completion or termination of employment. System accounts for departing personnel will be deactivated promptly, and the PI will be notified. Participant identity will remain confidential after trial completion and publication.

#### 2.12.2. Protocol Amendments and Deviations

Protocol amendments are changes to the ethics-approved protocol required for scientific, operational, or safety reasons. Amendments will be submitted to the ethics committee for approval before implementation. Participants or legal representatives will be informed of amendments affecting consent, safety, or continued participation and will sign an updated consent form when required. Protocol deviations will be classified as serious or non-serious according to their potential impact on participant rights, safety, health, or data integrity. Deviations will be documented, assessed for impact, corrected when possible, and reported to the ethics committee according to severity and institutional requirements.

#### 2.12.3. Dissemination

Trial results will be submitted to a peer-reviewed journal and reported in the trial registry according to registry requirements. Findings will be presented at scientific meetings when appropriate. A plain-language summary may be prepared for participants and families. Authorship and contributor roles will follow International Committee of Medical Journal Editors criteria and journal policies. Professional medical writers, if used, will be acknowledged and their role disclosed.

### 2.13. Patient and Public Involvement

No formal patient or public advisory panel was included in the initial protocol development. During the feasibility trial, the team will collect structured feedback from participants or legal representatives, where feasible, regarding the clarity of study information, the consent process, acceptability of oral treatment, and follow-up burden. This feedback will be used to refine the informed-consent materials and workflow for a future confirmatory trial.

### 2.14. Details of GenAI Tool Use

During manuscript preparation, ChatGPT (OpenAI, Sol 5.6) was used to assist with refining the English-language description of the study protocol. All trial procedures, scientific statements, and references were critically reviewed and verified by the authors. No participant-level clinical data were generated or analyzed using this tool.

## 3. Discussion

This protocol describes a randomized, double-blind, placebo-controlled feasibility trial of oral coenzyme Q10 in acute Stanford type A aortic dissection. Emergency surgical repair remains the standard life-saving treatment; the trial is restricted to patients who decline surgery after standard surgical counseling and is not intended to position coenzyme Q10 as an alternative to definitive operative treatment. The study is motivated by an unmet need in this highly selected population and by preclinical evidence implicating mitochondrial dysfunction and smooth muscle cell phenotypic switching in TAAD progression and rupture. By focusing on feasibility while retaining 14-day all-cause mortality as the primary exploratory clinical outcome, the study is designed to determine whether recruitment, consent, randomization, blinded treatment delivery, follow-up, endpoint assessment, and safety monitoring are operationally achievable in a high-risk emergency population.

The design has several strengths. Randomization and allocation concealment will reduce selection bias; matched placebo and double blinding will reduce performance and ascertainment bias; objective clinical, imaging, and laboratory outcomes will improve outcome reliability; and CRO/CRA-based monitoring, EDC validation, and data-management procedures will support conduct and data quality. The detailed harms framework aligns with the SPIRIT 2025 emphasis on harms assessment and intervention description.

The main limitations are the small sample size, single-center setting, short protocol-defined follow-up, and enrolment of only patients who decline surgery. This highly selected population limits generalizability and may introduce selection and survivorship bias; the reason(s) for refusing surgery, decision-making capacity, use of a legally authorized representative, and crossover to surgery will therefore be recorded prospectively. The 14-day follow-up is appropriate for the acute feasibility objective and the 14-day intervention but is insufficient to evaluate sustained survival, late aortic remodeling, or long-term safety. These longer-term outcomes should be incorporated into the subsequent definitive trial. Contemporary TAAD care spans acute diagnosis and treatment through post-dissection recovery and rehabilitation [18], underscoring the importance of longer-term evaluation in future studies. Treatment-effect estimates from this small feasibility trial will remain imprecise and should be interpreted as hypothesis-generating.

## 4. Conclusions

This randomized, double-blind, placebo-controlled feasibility trial will determine whether recruitment, treatment delivery, follow-up, outcome assessment, and safety monitoring are practicable in adults with acute Stanford type A aortic dissection who decline surgery. The resulting feasibility, safety, and exploratory event-rate estimates are expected to refine endpoint selection, sample-size planning, and operational procedures for a future confirmatory randomized trial of coenzyme Q10.

## Figures and Tables

**Table 1:** Eligibility criteria for trial participants.

Inclusion Criteria	Exclusion Criteria
Age ≥ 18 years. Stanford type A aortic dissection confirmed by aortic computed tomography angiography (CTA). Symptom onset within 24 h. The patient or legally authorized representative declines emergency surgical repair after standard surgical counseling. The patient or legal representative can understand the study purpose, procedures, potential benefits, and risks, voluntarily agrees to participate, and signs written informed consent. The patient or legal representative agrees to cooperate with study procedures and provide personal information and study-generated data for research analysis.	Irreversible organ, limb necrosis, or organ/limb failure. Severe acute respiratory distress syndrome, defined as acute or progressive dyspnea within 1 week of a known insult, bilateral infiltrates on chest X-ray or CT not fully explained by effusion/atelectasis/nodules, respiratory failure not fully explained by cardiac failure or fluid overload, and PaO_2_/FiO_2_ ≤ 100 mmHg. Blood gas lactate > 20 mmol/L. Hematochezia. Left ventricular ejection fraction < 25% on echocardiography. Hemoglobin < 30 g/L. Heart rate < 30 beats/min or systolic blood pressure < 60 mmHg not correctable by heart-rate- or blood-pressure-increasing medications. Persistent coma for >2 h. Any type of aortic dissection diagnosed before the first onset of symptoms for the current episode. Any prior cardiac or aortic surgery. Trauma-related aortic dissection. Pregnancy or lactation. Known allergy to coenzyme Q10. Coenzyme Q10 use within the previous 2 weeks. Inability to cooperate because of subjective factors such as lack of trust in medical staff or objective factors such as delirium or Alzheimer’s disease. Participation in another scientific study before enrolment without completion/withdrawal from that study.

**Table 2:** Study intervention, comparator, administration schedule, and concomitant care.

Group	Intervention	Administration	Duration	Concomitant Care
Coenzyme Q10 group	Coenzyme Q10 tablets. The marketed tablet strength is 10 mg; the protocol dose is 100 mg per administration.	100 mg orally, three times daily (tid).	14 days. First dose on Day 0; last planned dose on Day 13.	Same guideline-directed supportive care in both groups, including heart-rate and blood-pressure control, analgesia, and clinically indicated treatment of comorbidities/complications (Section 2.5.3).
Placebo group	Matched placebo tablets identical to active drug in size, shape, color, appearance, and odor.	100 mg orally, three times daily (tid).	14 days. First dose on Day 0; last planned dose on Day 13.	Same guideline-directed supportive care as the coenzyme Q10 group.

**Table 3:** Feasibility, exploratory efficacy, safety outcomes, and assessment time points.

Outcome Domain	Specific Outcome	Measurement Variable and Aggregation	Assessment Time Point(s)
Primary feasibility outcome	Recruitment feasibility	Achievement of the planned recruitment target, defined as randomization of 60 participants within 12 months after recruitment initiation. The numbers screened, eligible, consented, and randomized and the recruitment rate will also be summarized descriptively.	At the end of the 12-month recruitment period or when 60 participants have been randomized, whichever occurs first.
Primary exploratory efficacy outcome	14-day all-cause mortality	The proportion of participants who die from any cause by the final scheduled assessment, excluding death attributable to a tumor, natural disaster, or accidental causes unrelated to TAAD. Earlier all-cause mortality will also be summarized descriptively.	Study Day 14 (treatment Day 15; final visit window +2 days); descriptive interim assessments on Study Days 1, 2, and 7.
Exploratory efficacy outcomes	Aortic rupture death	Proportion with death directly attributable to aortic rupture confirmed by echocardiography, aortic CTA, aortic MRA, or other objective imaging.	Study Days 1, 2, 7, and 14.
Exploratory efficacy outcomes	False-lumen thrombosis	Proportion with partial or complete thrombosis of the false lumen in any segment involved by dissection, confirmed by aortic CTA.	Study Days 7 and 14.
Exploratory efficacy outcomes	Cardiac tamponade	Proportion with clinical manifestations of tamponade supported by echocardiographic findings, including chamber collapse, abnormal inferior vena cava findings, swinging heart, pericardial effusion, respiratory variation in transvalvular flow, abnormal hepatic venous flow, or septal shift.	Study Days 1, 2, 7, and 14.
Exploratory efficacy outcomes	Worsening pericardial effusion	Proportion with new pericardial effusion or worsening effusion compared with baseline on echocardiography.	Study Days 7 and 14.
Exploratory efficacy outcomes	Organ malperfusion syndromes	Proportions with cerebral, mesenteric, limb, renal, coronary, or spinal cord malperfusion, defined by arterial involvement on CTA plus protocol-specified symptoms, events, or laboratory/imaging findings for each organ territory.	Study Days 1, 2, 7, and 14; renal malperfusion is specifically assessed on Study Days 7 and 14.
Safety outcomes	Adverse events (AEs), serious adverse events (SAEs), adverse drug reactions (ADRs), and suspected unexpected serious adverse reactions (SUSARs)	Number and proportion of participants with each event type, classified by severity, seriousness, causality, action taken, treatment, outcome, and relationship to study drug.	From signing informed consent through the end of the study; extended follow-up after early withdrawal as defined in the protocol.
Laboratory safety outcomes	Hematology, biochemistry, thrombosis markers, myocardial injury markers, coagulation, blood gas, and pregnancy test when applicable	Change from baseline and final values for prespecified laboratory measurements, summarized by group.	Baseline screening and scheduled follow-up as shown in the participant timeline.
Secondary feasibility outcomes	Screening/eligibility yield, treatment adherence, follow-up completion, dropout, missing data, endpoint assessment feasibility, and candidate endpoint event rates	Counts, proportions, rates, and descriptive summaries by group and overall, as applicable.	Throughout recruitment and at final follow-up.

**Table 4:** Schedule of enrolment, intervention, assessments, and follow-up.

Study Procedure	Screening Days −13 to −1	Day 0	Day 1	Day 2	Day 7	Day 13	Follow-Up Day 14 (+2)	Early Withdrawal
Written informed consent	X							
Demographics and personal information	X							
Medical history	X							
Physical examination	X							
Aortic CTA	X				X		X	(X)
Echocardiography	X				X		X	(X)
Hematology	X						X	(X)
Blood biochemistry	X						X	(X)
Coagulation tests	X							
Thrombosis markers	X						X	(X)
Myocardial injury markers	X						X	(X)
Blood gas analysis	X							
Pregnancy test for women of childbearing potential	X							
Eligibility assessment	X							
Randomization		X						
Study medication		X	X	X	X	X		
Survival follow-up		X	X	X	X		X	(X)
Efficacy endpoints		X	X	X	X		X	(X)
AE/SAE collection	X	X	X	X	X	X	X	(X)
Concomitant medication collection	X	X	X	X	X		X	(X)

## Data Availability

No new participant-level data were generated or analyzed for this study protocol. After completion and publication of the trial, de-identified participant-level data, the data dictionary, and statistical code may be made available upon reasonable request, subject to approval by the principal investigator, applicable ethics requirements, and a data-use agreement.

## Supplementary Materials

The following supporting information can be downloaded at: https://www.mdpi.com/article/10.3390/jcm15208103/s1, Table S1: SPIRIT 2025 Checklist.

## Abbreviations

The following abbreviations are used in this manuscript:

AD	Aortic dissection
ADR	Adverse drug reaction
AE	Adverse event
APTT	Activated partial thromboplastin time
BUN	Blood urea nitrogen
CK-MB	Creatine kinase isoenzyme MB
CRA	Clinical research associate
CRC	Clinical research coordinator
CRF	Case report form
CRO	Contract research organization
CTA	Computed tomography angiography
EDC	Electronic data capture
GCP	Good Clinical Practice
IRT	Interactive response technology
LVEF	Left ventricular ejection fraction
MRA	Magnetic resonance angiography
MRI	Magnetic resonance imaging
PI	Principal investigator
SAE	Serious adverse event
SAP	Statistical analysis plan
SMO	Site management organization
SPIRIT	Standard Protocol Items: Recommendations for Interventional Trials
SUSAR	Suspected unexpected serious adverse reaction
TAAD	Type A aortic dissection
tid	Three times daily
