# Supplementary material for: Coenzyme Q10 for Acute Stanford Type A Aortic Dissection: Protocol for a Prospective, Randomized, Double-Blind, Placebo-Controlled, Single-Center Feasibility Trial
Source: J Clin Med. 2026 Oct 19;15(20):8103. doi: 10.3390/jcm15208103 (PMC13706217; doi:10.3390/jcm15208103)
Supplement: Supplementary file 1 [file jcm-15-08103-s001.zip › jcm-4577268-supplementary.pdf]

Supplementary Table S1. SPIRIT 2025 Checklist

Coenzyme Q10 for Acute Stanford Type A Aortic Dissection: Protocol for a Prospective, Randomized, Double-Blind, Placebo-Controlled, Single-Center Feasibility Trial

SPIRIT 2025 item	Where addressed in this draft	Items requiring study-team confirmation before submission
1a Title	Title page	
1b Structured summary	Abstract	
2 Protocol version	Administrative information; trial status	
3a Contributors	Administrative information	Author names, affiliations, ORCID IDs, and roles
3b Sponsor contact	Administrative information	Sponsor contact details
3c Sponsor/funder role	Administrative information	Funding source and role
3d Oversight groups	Administrative information; monitoring	Names/charter of oversight groups if applicable
4 Trial registration	Administrative information; trial status	Registry name, number, URL, registration date
5 Protocol/SAP access	Administrative information	Repository or request mechanism
6 Data sharing	Administrative information; declarations	Final institutional data-sharing policy
7a Funding	Administrative information; declarations	Funding details
7b Conflicts	Administrative information; declarations	Completed author disclosures
8 Dissemination	Administrative information; ethics and dissemination	
9a Background/rationale	Introduction	
9b Comparator choice	Introduction	
10 Objectives	Introduction	
11 Patient/public involvement	Methods	
12 Trial design	Methods	
13 Setting	Methods	
14a Participant eligibility	Eligibility criteria	
14b Site/personnel eligibility	Eligibility criteria for sites and deliverers	
15a Intervention/comparator	Intervention and comparator	
15b Discontinuation/modification	Intervention section	
15c Adherence	Intervention section	
15d Concomitant care	Intervention section	

16 Outcomes	Outcomes table	
17 Harms	Harms; safety oversight	
18 Timeline	Participant timeline table	
19 Sample size	Sample size	
20 Recruitment	Recruitment	
21a-b Sequence generation	Assignment of interventions	
22 Allocation concealment	Assignment of interventions	
23 Implementation	Assignment of interventions	
24a-c Blinding	Blinding section	
25a Data collection	Data collection methods	
25b Retention	Retention and follow-up	
26 Data management	Data management	
27a-d Statistical methods	Statistical methods	Final SAP details
28 Interim/stopping	Interim analyses and stopping rules	
29 Monitoring	Trial monitoring	Confirm whether DSMB/independent medical monitor is required
30 Harms reporting	Harms reporting and safety oversight	
31 Confidentiality	Confidentiality	
32a-b Consent	Informed consent	
33 Ethics	Ethics approval	Ethics approval number/date
34 Ancillary/post-trial care	Risk-benefit considerations; harms reporting	Clarify participant compensation/insurance and post-trial care policy
